# TEACHING IN MEDICAL RESIDENCY IN TIMES OF COVID-19

**DOI:** 10.1590/1984-0462/2020/38/2020334

**Published:** 2020-11-27

**Authors:** Mário Cícero Falcão, Camila Dal Piccolo Pracchia Fonseca, Gabriel Vecchi Danti

**Affiliations:** a Department of Pediatrics, Faculty of Medicine, Universidade de São Paulo, São Paulo, SP, Brazil.; b Instituto da Criança of Hospital das Clínicas, Faculty of Medicine, Universidade de São Paulo, São Paulo, SP, Brazil.

The content of the Medical Residency Program in Neonatology of Instituto da Criança of Hospital das Clínicas, Faculty of Medicine of Universidade de São Paulo, underwent a profound transformation of its scenario before the current SARS-CoV-2 pandemic.

Before the pandemic, the referred content used to be taught in two monthly meetings, one for 1^st^ year residents (20 resident doctors) and one for 2^nd^ year residents (20 resident doctors), totaling ten annual meetings.

The dynamics of these meetings included the presentation and discussion of a clinical case at the Neonatal Center, the Intensive Care Center 2 or the University Hospital by a resident doctor; and the theoretical approach on the case, with its definitions, prevalence, pathophysiology, treatment, and prevention , by another resident doctor.

The main topics addressed by the three neonatal centers were:

- Neonatal Center: extreme preterm newborns, persistence of the ductus arteriosus, severe malformations, heart failure, early sepsis, and hypoxic-ischemic encephalopathy;
- Neonatal Intensive Care Center 2: persistent pulmonary hypertension, congenital diaphragmatic hernia, late-onset sepsis, lung infections, gastroschisis, hydroelectrolytic disorders and nutrition in critically ill newborns;
- University Hospital: intracranial hemorrhages, kidney failure, meconium aspiration syndrome, and necrotizing enterocolitis.

These meetings were held by the two preceptor doctors and by two to three doctors from the Neonatology team, the latter being responsible for reinforcing the main relevant considerations on the topic in question.

The presence of resident doctors at these meetings was between 60 and 70%. The justification for this percentage not being greater was post-shift leave, medical emergency in some of the sectors, and vacations.

With the coronavirus pandemic (COVID-19), these meetings were suspended in mid-March 2020, for the safety of all, as well as other activities specific to residents. For one month, residents had very little or no theoretical activity.

Given the moment of much apathy, alternative proposals were suggested in remote meetings, among them, remote learning.

Remote learning is an option when in-person learning is not possible, as it is the case during the COVID-19 pandemic. It is an adaptation of distance learning, but without rigid tools, that is, without systematization of didactic content, form of presentation, and without prior training of tutors. Although this is a temporary adaptation in the face of a crisis, its implementation is quick, effective and, thus, it satisfies the aspirations of our residents. The adoption of remote education also implies a review of educational objectives, with time scaling, because the time to assimilate content in a virtual environment is longer than what is needed in person.^1^

An initiative of the two preceptor doctors and two doctors of the Neonatology team, a first virtual meeting was held in the second half of April 2020, using Google Meet platform, with all the 1^st^ and 2^nd^ year residents, addressing a theme suggested by one of the preceptors.

Although the referred platform is intuitive and easily usable, the link to a Google Meet tutorial prepared by the Medical Education Development Center of the Faculty of Medicine of Universidade de São Paulo was passed on to the team’s doctors and residents who were not familiar with the platform.^1^ ^,^ ^2^ The main topics covered are instructions on how to enter the platform, participate in the meeting, invite people and start (synchronous teaching) and record (asynchronous teaching) the presentation on Google Drive, to be made available later. The tutorial also reminds that, when starting a presentation, the presenter only sees your screen and not who is watching it. In addition, it also guides how to end the presentation and start living chat with meeting participants.

We must be very attentive to the chat because teaching depends on interaction based on dialogue. In the virtual environment, this is more difficult to achieve due to the lack of eye contact. Thus, in remote education, chat is an important channel of dialogue to improve learning.

The tutorial also highlights how to obtain good video quality: choose a place without noise and with adequate lighting, emphasizing that natural light is ideal (if impossible, the source must be positioned next to the camera); keep in mind that light backgrounds need more contrast and, darker, more luminosity; be aware of your speech pace; always maintain eye contact with the camera and, finally, be authentic.

Adherence to the pilot project was excellent, going beyond our expectations. We believe that this adhesion occurred due to the peculiar moment that we were going through, before an unknown, serious illness, leading the residents to conditions of depression and apathy.

In view of these facts, a program of theoretical content was created, in the form of livestreams, in the same platform, divided into several activities, namely: discussion of clinical cases, with presentation of theoretical content (similar to what we already did before the pandemic started), discussion of articles with emphasis on methodology, and lectures. These activities are the responsibility of selected resident doctors and doctors from the Neonatology team, who voluntarily joined the program, together with the preceptors. Lectures are taught by the team and, if they are taught by a resident, someone on the team is responsible for validating content.

Among the topics addressed in remote learning, those related to perinatal nutrition stand out, namely: “maturation of digestive function, gastrointestinal development, and digestion and absorption of macronutrients”, “neuronutrients”, “vitamin D, calcium, and phosphorus metabolism in the neonatal period”, “bioactive components of human milk”, “ homologous and heterologous additives in human milk”, “technological advances in infant formulas”, “bases of enteral nutrition in preterm newborns”, “use of prebiotics in premature infants”, “difficulties in the diagnosis of gastroesophageal reflux disease in the neonatal period”, “ nutritional therapy in newborns with congenital heart disease”, “indications and benefits of hypercaloric formulas”, “chylothorax approach”, “nutritional management in gastrointestinal malformations”, “necrotizing enterocolitis and nutrition”, “nutrition and bronchopulmonary dysplasia”, “nutritional challenges in aerodigestive dysfunction”, and “nutritional follow-up of very low-weight newborns”. None of these themes were addressed in in-person meetings with neonatology residents at Instituto da Criança of Hospital das Clínicas, Faculty of Medicine of Universidade de São Paulo.

Online learning can be a convenient way to maintain an adequate education in times of social distancing, but it requires more effort from instructors. One learns from experience, and online teaching requires more preparation time, perhaps more than we might imagine.

With online teaching, more people are reached than with traditional in-person methods. The COVID-19 pandemic is a moment to innovate, and we can discover new ways to teach online. Best of all: although it can be very laborious, almost everything that is produced is reusable, resulting in a very good infrastructure to be replicated.^2^

The livestreams of the theoretical program of neonatology residents are structured according to the guidelines of Harvard Business Publishing, published in 2017,^3^ to prepare, teach, and call participants’ attention online, namely: to know the online learning management system chosen before starting your presentation; making the learning management system look professional and inviting; remembering that electronic tools leave traces and using that to your advantage; preparing the content in advance and reviewing it; emphasizing the importance of questions and inquiries about the topic in question; and congratulating students’ engagement and participation.

Before the pandemic, residents had a monthly meeting. After the implementation of the livestreams program, these activities started to happen from two to three times a week, that is, in one month, we have the same number of meetings we had in a year before the pandemic.

Frequency of residents at these meetings has increased significantly, as they can access them remotely, even from their mobile device. In addition, Google Meet platform allows recording the activity, so that it can be watched later, increasing the presence of residents to classes, case discussions, etc. It is logical that watching the recording implies loss, because residents are not able to have their questions answered live in Google Meet chat.

Thus, in the face of the COVID-19 pandemic, our residents did not lose the theoretical content of the Neonatology Medical Residency Program for their training, they only gained substantially.

Another interesting fact is that, given the reputation that the livestream program has achieved, other doctors on the team volunteered to carry out activities with residents. There are also requests to attend these meetings by former residents of our program, who learned about the didactic activities through various groups.

In conclusion, we emphasize that remote learning is a very inexpensive program, because the platform use is free, feasible at a distance, and can be performed by all medical specialties, all that is needed is having the willingness and availability to implement it. In addition, we believe that, after the COVID-19 pandemic, this type of learning will also be used in a hybrid model, with in-person and online meetings. We intend to carry out a questionnaire with all residents to evaluate this teaching tool. Based on its results, we can adopt this model for theoretical learning for residents.
